# Proton Therapy in Head and Neck Cancer Treatment: State of the Problem and Development Prospects (Review)

**DOI:** 10.17691/stm2021.13.4.08

**Published:** 2021-08-28

**Authors:** K.B. Gordon, D.I. Smyk, I.A. Gulidov

**Affiliations:** Senior Researcher, Proton Therapy Department; A. Tsyb Medical Radiological Research Centre — Branch of the National Medical Research Radiological Centre of the Ministry of Health of the Russian Federation, 4 Koroleva St., Kaluga Region, Obninsk, 249036, Russia; Junior Researcher, Proton Therapy Department; A. Tsyb Medical Radiological Research Centre — Branch of the National Medical Research Radiological Centre of the Ministry of Health of the Russian Federation, 4 Koroleva St., Kaluga Region, Obninsk, 249036, Russia; Professor, Head of the Proton Therapy Department; A. Tsyb Medical Radiological Research Centre — Branch of the National Medical Research Radiological Centre of the Ministry of Health of the Russian Federation, 4 Koroleva St., Kaluga Region, Obninsk, 249036, Russia

**Keywords:** proton therapy, head and neck cancer, reirradiation therapy

## Abstract

Proton therapy (PT) due to dosimetric characteristics (Bragg peak formation, sharp dose slowdown) is currently one of the most high-tech techniques of radiation therapy exceeding the standards of photon methods.

In recent decades, PT has traditionally been used, primarily, for head and neck cancers (HNC) including skull base tumors. Regardless of the fact that recently PT application area has significantly expanded, HNC still remain a leading indication for proton radiation since PT’s physic-dosimetric and radiobiological advantages enable to achieve the best treatment results in these tumors.

The present review is devoted to PT usage in HNC treatment in the world and Russian medicine, the prospects for further technique development, the assessment of PT’s radiobiological features, a physical and dosimetric comparison of protons photons distribution. The paper shows PT’s capabilities in the treatment of skull base tumors, HNC (nasal cavity, paranasal sinuses, nasopharynx, oropharynx, and laryngopharynx, etc.), eye tumors, sialomas. The authors analyze the studies on repeated radiation and provide recent experimental data on favorable profile of proton radiation compared to the conventional radiation therapy.

The review enables to conclude that currently PT is a dynamic radiation technique opening up new opportunities for improving therapy of oncology patients, especially those with HNC.

## Introduction

Head and neck cancers (HNC) are among the ten most common tumors both in Russia and worldwide [1, 2]. Radiation therapy is widely used as an independent technique to treat such diseases, as well as an adjuvant therapy after surgery. Moreover, due to known anatomic features, HNC are difficult for radiation. The basic radiation method used in a routine practice is photon therapy. The latest advances in this sphere — intensity-modulated radiation therapy (IMRT) and volumetric modulated arc therapy (VMAT) — enable to significantly improve the radiation accuracy and conformity that has noticeably decreased the load on the surrounding healthy organs and tissues. As a result, the frequency of complications in these tumors — xerostomia and trismus — has been managed to reduce significantly [3, 4].

However, the obtained dosimetric advantage resulted in the frequency and intensity growth of acute radiation responses (mucositis, radioepidermites, general weakness, nausea, lymphocytopenia, alopecia). The responses occur due to an increased volume of healthy tissues under low-dose radiation in the field modulation, as well as an inhibitory effect of photons on lymphocyte count and function [3, 5]. Considering the physics of dose distribution, it is becoming clearer that a standard photon therapy is likely to have achieved its technological limit, and its further improvement is unlikely to affect the radiation quality.

Accordingly, proton therapy (PT) arouses particular interest as it is one of the most sparing radiation techniques, which enables to reduce toxicity and, subsequently, improve oncology treatment outcomes. Unique physical characteristics of charged particles (Bragg peak) enable to achieve better dose distribution in healthy body tissues compared to photon therapy, and it significantly reduces the rate and intensity of radio-induced responses.

## Technical and dosimetric aspects of proton therapy

A proton beam can be formed using two basic techniques: passive scattering and active scanning. In passive scattering, a proton beam is distributed in space using scattering foil, and its form is given by an aperture, approximately the same as it is performed in 3D conformal photon therapy. Depth dose distribution, in this case, is modeled by compensators. Compared to an active scanning beam, a passive scattering technique is currently outmoded, since it has the worst dose distribution characteristics, and requires field-forming devices, as well as it is characterized by the formation of secondary neurons.

IMPT is based on magnetic properties of particles. In a cyclotron or a synchrotron, there generates a thin beam of protons with particular energy necessary to achieve the tumor depth. A beam path deviates through the use of magnets, and in this way, the protons gradually fill the whole volume of the target irradiation. Currently, IMPT is the most precise, widely used PT type.

The reverse side of proton spatial distribution advantages is their sensitivity to physical and geometrical errors, which are important to take into account when preparing and during a course of treatment [6–8]. For a precise dose calculation and treatment planning optimization, Monte Carlo algorithm is used in modern planning systems instead of formerly used pencil-beam methods. In cases of patient’s weight loss, a target geometry change, and in tumors of specific localizations (e.g. sinonasal lesions), as well as in the situations when the loads on risk organs are close to boundary values, or slightly exceeding them, an additional verification can be needed. For example, Gunn et al. [9] estimated a PT planning process in 50 patients with HNC as follows: 38% of cases required planning correction due to weight loss or tumor form change; in one case, a new planning was to be performed twice.

A similar “grey” zone is a real value of relative biological effectiveness (RBE) of protons indicating the difference of an effective dose of protons and photons. Currently, proton RBE is accepted to be equal to 1.1, although an increasing number of researchers believe that the value is variative and can be higher when it is very close to Bragg peak [10]. Some PT centers, in dosimetric calculation, take into consideration both: a physical dose, and also the coefficients of linear energy transfer, and calculate an individual RBE [11].

The dosimetric advantages of protons over photons in the treatment of nasopharyngeal, sinonasal, and oropharyngeal tumors were compared as early as in 1989–1992 [12, 13]. So far, there have been published a lot of articles comparing proton therapy with photon techniques: 3D conformal therapy, IMRT, and VMAT. The papers [14–17] have confirmed a marked dose reduction using PT compared to IMRT in healthy tissues in patients with uni- and bilateral oropharyngeal tumors, both in a postoperative period and in reirradiation. The study [18] represents the comparison of 25 PT plans with IMRT in patients having been treated for oropharyngeal cancers. The analysis of the findings showed that the doses falling within the anterior and posterior oral cavity, and inferior pharyngeal constrictors, the esophagus, and other masticatory structures, brain stem, cerebellum, and other CNS organs, in PT plans were significantly lower.

As already mentioned above, radiation therapy in patients with HNC results in early and late responses and complications. Most commonly, during therapy, patients develop mucositis, pain syndrome, weakness, shift in tastes, dehydration, weight loss, vomiting, local dermatitis. The majority of the mentioned responses directly depend on a received dose with a tendency for toxicity enhancement or a complete organ dysfunction at large doses and target volumes. The collected data enabled to assess the risk of responses and predict their occurrence by studying clinical and dosimetric characteristics. For example, there are known average radiation loads in xerostomia [19, 20], dysphagia including that one leading to tube feeding [21, 22], hypothyroidism [23], laryngeal edema [24], nausea [25], and other early radiation complications [26]. Most of the data on radiation dose limits on risk organs have been recently included in the recommendations by QUANTEC, and these recommendations are to be adhered to in clinical practice [27].

PT has an expected advantage in the form of toxicity frequency reduction [28]. Thorough selection of patients based on comparison models was proved to be able to reduce severe radiation complication risks, and also decrease rehabilitation costs [29, 30]. It should be noted that the data on tissue tolerance to radiation have been obtained primarily for photon techniques so far. Moreover, the study by Blanchard et al. [31] has shown that most of the existing restrictions can be used for PT planning; however, the question needs to be further analyzed.

## Proton therapy in skull base tumors

Historically, one of the first extracerebral targets in HNC treated by PT was skull base chordoma. The localization and tumor morphological type are associated with very close position to critical structures. For successful local control over chordoma and chondrosarcoma, the arrangement of high doses of ionizing radiation is necessary. It is extremely difficult to expose to radiation the area using photon therapy without an increased risk of complications, therefore, some experts concur that photon therapy is useful only if PT is not performed [32]. Moreover, in the majority of cases, radical surgery of such localized lesions renders difficult due to a complex anatomic position and the tumor extent. PT, as a rule, enables to use efficient doses, not exceeding maximum radiation exposure on the surrounding structures. As early as in 1999, Munzenrider et al. [33] studied the treatment results of 519 patients with chordomas and chondrosarcomas treated with a combination of photon and proton therapy, a total dose being up to 66–83 isoGy. A five-year recurrence-free survival rate after therapy was 73% in patients with chordomas, and 98% — with chondrosarcomas. A five-year total survival rate was 80 and 91%, respectively. It is significant that the frequency of severe complications (stage IV–V) was low. So, only 3 patients (0.5%) died of brain stem lesions, and 8 patients (1.5%) suffered from temporal lobe lesions, hearing loss, intracranial neuropathy, and endocrinopathy.

High figures of local control in PT were confirmed by other researches as well [34, 35]. Five- and ten-year total toxicity rate, stage III–V, in recent studies published on the topic also does not exceed 10% [36–38].

## Proton therapy in head and neck cancers

A standard procedure used to treat nasal and sinonasal tumors is surgery and further radiotherapy combined with or without chemotherapy depending on a disease stage. In this case, PT enables to achieve high total doses preserving patients’ good life quality. The Appendix represents the results of PT studies of HNC including this localization. The most clinical observations are included in the study by Resto et al. [39] published in 2008. The investigation involved 102 patients who underwent treatment from 1991 to 2002. Most patients at the first stage underwent surgery followed by combined photon-proton therapy. The surgery radicality had an effect, primarily, on total survival rate (p=0.02), progression-free survival (p=0.009), and the risk of delayed-effect risk (p=0.03). In a group of patients with total tumor resection, local control index was 95%, in those with partial resection — 82%, while in patients who had just undergone biopsy — 87% (p=0.32). In general, treatment failures were primarily due to a delayed progression: 30% of patients within a five-year period were found to have some metastases.

The studies mentioned in the Appendix (Table 1) involve a great number of patients with a high survival rate; however, the research data are compared with historical groups of patients who had received photon therapy. Just one study [40] involved the controls who had received photon therapy. The authors stated that in IMRT group they frequently had to use analgesics and feeding tubes. However, it should be noted that in the group there were more patients with nasopharyngeal tumors who required preventive radiation of cervical lymph nodes. A significant observation (see Appendix, Table 1) is the following: there was good survival rate and local control in patients with nasal mucosa melanoma who underwent an independent PT course, and the moderate frequency of late radiation complications (stage III–IV) in them was 20% that was significantly lower compared to photon group therapy [41–43].

**Table 1:** Nasal and sinonasal tumors

Studies	Study type	Study period (years)	n	Technique	PhT	PCT (%)	OF (%)	Morphology	Observation median (months)	Results	Toxicity
Resto et al. [39]	Retro	1991-2002	102	PSPT		4	100	Different	61	5-year: LC - 95, 82, and 87%; OS - 90, 53, and 49% in groups R0, R1, and biopsy	Not evaluated
Russo etal. [71]	Retro	1991-2008	54	PSPT	-	39	69	HNSCC	82	5-year: LRC - 73%; OS - 47%	Stage III — in 9; IV — in 6
Nakamura et al. [72]	Retro	1999-2012	42	PSPT	-	26	0	Esthesioneuroblastoma	69	5-year OS/PFS: 100/80% — group A; 86/65% — group B; 76/39% — group C	Stage lll-IV — in 6: visual loop — 4; liquorrhea — 1; cataract — 1
Takagi et al. [73]	Retro	2002-2012	40	PSPT		0	0	Adenocarcinoma	38	5-year: OS - 63%; PFS - 30%; LC - 76%	Stage lll-V — in 36 (26%): III — in 24 (osteonecroses); IV — in 9 (vision loss); V — in 3 (radiation ulcers)
Demizu etal. [41]	Retro	2003-2011	33	PSPT	-	0	0	Melanoma	18	2-year: LC-71%; OS-44%	Stage lll-IV — in 3: cataract, mucositis, and pain
Linton et al. [74]	Retro	2004-2012	26	PSPT		0	77	Adenocarcinoma	25	2-year: LC - 95%; OS - 93% (without previous radiation)	Late toxicity, stage III — in 2; IV — in 1; V — in 1 (after a repeated course)
Fuji et al. [42]	Retro	2006-2012	20	PSPT	-	0	0	Melanoma	35	3-year: OS - 68%; PFS - 60%	Stage IV — in 1 (visual loop)
Dagan et al. [75]	Retro	2007-2013	84	PSPT		75	74	Different	32	3-year: LC — 83%; no metastases — 72%; OS - 68%	Stage lll-V (24%): cerebral necrosis — in 1; osteonecrosis/soft tissue necrosis — in 7; 3 fatal cases
Zenda et al. [43]	Pro	2008-2012	32	PSPT	-	0	0	Melanoma	36	1-year: LC-76%; 3-year: OS - 46%; PFS - 36%	Stage >lll — none
McDonald et al. [40]	Retro	2010-2014	40	PSPT	+	75	—	Different	—	—	In IMRT, tubes and narcotics were more frequently used
Patel et al. [44]	Retro	1975-2013	286	PSPT/ C-ions	+	—	—	Different	38	OS >5 years in hadron therapy group (p=0.0038) and in higher observation median (p=0.037), the same as PFS (p=0.0003)	

In 2014, Patel et al. [44] presented a meta-analysis of hadron therapy involving the treatment data on 286 patients with various morphological tumor types of sinuses, from 1975 to 2013. The observation median was 38 months in the hadron therapy and photon therapy groups. Charged-particle radiation group was found to have significantly higher five-year survival rate (p=0.0038) and a five-year recurrence-free rate (p=0.0003). Five-year loco-regional control in both groups was nearly the same (p=0.79), but it grew in a hadron therapy group as the follow-up increased (p=0.031). Regardless of the fact that most studies involved in the meta-analysis were retrospective, and due to a large number of observations and repeatability of the results within nearly 40 years, the researchers concluded particle-charged radiotherapy (chiefly, PT) to be definitely a high-efficient therapy option for patients with nasal and sinonasal tumors.

One of the first researches describing PT usage to treat pharyngeal tumors was published by Slater et al. [45] in 2005. The study included treatment results of 29 patients with tumors (stage II–IV), who underwent a combined therapy: 3D conformal radiotherapy (up to total focal dose equal to 50.4 Gy) and passive scattering PT (boost 25.5 isoGy) in the period from 1991 to 2002. The observation median was 28 months, and the authors reported good five-year loco-regional control and recurrence-free survival rate (88 and 65%, respectively). The radiation complication (stage III, and higher) rate during the first two years was less than 16%.

It should be noted that most studies on PT used in the tumors of oropharynx, nasopharynx, and nasal cavity are prospective (see Appendix, Table 2).

**Table 2:** Oropharynx, nasopharynx, and nasal cavity tumors

Study	Study type	Study period (years)	n	Technique (dose)	PhT	PCT	Localization	Observation median (months)	Results	Toxicity
Slater et al. [45]	Retro	1991-2002	29	^60^Co (50.4 Gy); PSPT (25.5 isoGy)	—	—	Oropharynx	28	5-year: LRC — 88%; PFS - 65%	Stage >lll — in 16%
Takayama et al. [76]	Pro	2009-2012	33	PhT (36 Gy) + PSPT (28.6-39.6 isoGy)	-	+	Oral cavity	43	3-year: LC — 86.6%; LRC-83.9%; OS-87.0%	Stage >lll — none
Chan et al. [77]	Pro	2006-2011	23	PSPT (70 isoGy)	-	+	Nasopharynx	28	2-year: LRC-100%; OS-100%	Stage >lll — none
Lewis et al. [78]	Pro	2011-2013	10	IMPT (70 isoGy)	+	+	Nasopharynx	24	2-year: LRC-100%; OS - 88.9%	Fewer gastrostomas (p=0.02)
Gunn et al. [9]	Pro	2011-2014	50	IMPT (70 isoGy)	+	+	Oropharynx	30	3-year: LRC-91%; OS - 94.3%	Fewer gastrostomas and weight loss. Better life quality

Blanchard et al. in 2016 [46] published the results of a comparative study of two groups of patients treated for oropharynx cancer (using proton and photon therapy). The first group (n=50) with IMPT was compared with the controls (n=100) treated by IMRT. The observation median was 32 months. There were no significant differences in overall survival and recurrence-free survival, although in IMRT group the weight loss rate, as well as the necessity for feeding tubes within the first three months and a year were higher (p=0.05 and p=0.01, respectively).

Sio et al. [47] in their study in 2016 carried out the life quality analysis of patients with nasopharyngeal tumors after IMPT and found the technique to contribute to the reduction of post-radiation responses; the patients got through a rehabilitation period easier compared to those with photon therapy. Both patient groups with different treatment forms were found to have high loco-regional characteristics and survival rate within two years after therapy; in addition, the necessity for feeding tubes was low.

A prospective study by Hayashi et al. [48] considered the combination of PT with intra-arterial chemotherapy in the treatment of regional glosoncus in patients who refused surgery. The observation median was 43 months. Three-year local control, regional control, recurrence-free survival and overall survival rates were 86.6, 83.9, 74.1, and 87.0%, respectively. No osteoradionecrosis (stage III) was revealed; however, the authors found frequent caries cases (up to 30%).

## Proton therapy in orbital tumors and sialomas

One of PT advantages is high probability of preserving an organ or its function after radiation. In 2016, there were published the findings of a combination therapy (surgery and PT) in patients with periorbital tumors [49, 50]. The main endpoints of the study by Holliday et al. [50] were to save eye functions, oncologic result achievement, and a cosmetic effect. The analysis involved 20 patients, who underwent surgery at the first stage, and PT — at the second stage. 7 cases had lacrimal gland cancer, 10 patients — lacrimal sac or nasolacrimal canal tumors, and 3 patients — blepharoncus. Histologically, the tumors were adenocarcinomas and squamous cell carcinomas. The observation median was 27.1 months, but no local recurrences that would require reoperation were recorded. One patient was found to have regional progression, and metastases were detected in one patient. Most complications were the following: chronic lacrimation, and III stage keratopathy (15%). Four patients (20%) had visual loops.

PT is also an important modality in treating sialomas, which are known as radio-resistance, and they require large radiation doses for successful local control. Romesser et al. [51] studied, primarily, the advantages of passive scattering PT over IMRT; and found PT patients to have significantly lower frequency of taste sensation change (5.6 vs 65.2%), mucositis (16.7 vs 52.2%), and general weakness (11.1 vs 56.5%). The authors [52] reported great dosimetric differences of PT and IMRT in patients with parotid salivary gland tumors, in favor of PT.

## Proton therapy in reirradiation

It should be noted that a reduced integral radiation load in PT has certain prospects in reirradiation (see Appendix, Table 3). In 2016, Romesser et al. [53] represented a multi-center retrospective analysis, which involved 92 patients exposed to PT reirradiation using a passive scattered beam, from 2011 to 2014. During the first year, after therapy, loco-regional recurrence rate was 25.1%, while overall survival rate was 65.2%, and the recurrence rate of stage III–V complications was 14.1%. Phan et al. [54] in the same year represented the findings of proton reirradiation of 60 patients with HNC, and 45 of them were treated using IMPT: the recurrence-free rate and overall survival rate at the first year were 68.4 and 83.8%, respectively. Most notably, both research groups agreed that proton reirradiation is significantly less toxic compared to photon therapy.

**Table 3:** Reirradiation of head and neck tumors

Study	Study type	Study period (years)	n	Technique	PCT (%)	OF (%)	Morphology	Observation median (months)	Results	Toxicity
McDonald et al. [79]	Retro	2004-2014	61	PSPT	29	47.5	HNSCC (37), others (24)	29	2-year: LR —19.7%; OS - 32.7%	Stage III — in 8 (necroses); IV —in 4: blindness —1, necrosis — 1; V — in 3
Phan et al. [54]	Pro	2011-2015	60	PSPT (n=15) IMPT (n=45)	73	58	HNSCC (40), others (20)	13.6	1-year: LRS-68.4%; OS - 83.8%	Acute stage III (30%): tubes; late stage lll-V (16.7%)
Romesser et al. [53]	Retro	2011-2014	92	PSPT	39	39	HNSCC (52), others (40)	13.3	1-year: LRR-25.1%; OS - 65.2%	Late stage lll-V (14.1%)
Hayashi et al. [48]	Pro	2009-2013	25	PSPT	—	46	HNSCC	24	2-year: LR - 30%; OS - 46%	Stage IV — in 1

Notes: n — number of patients; PSPT — passive scattering proton therapy; OF — operation frequency; PCT — polychemotherapy application frequency; PhT — photon therapy; IMRT — intensity-modulated radiation therapy; LR — local recurrence; LC — local control; LRC — loco-regional control; OS — overall survival; LRS — local recurrence survival; PFS — progression-free survival; LRR — loco-regional recurrence; PINSCC — head and neck squamous cell carcinoma; retro — retrospective; pro — prospective.

## Proton therapy prospects

One of the most essential factors hindering PT development is high cost of proton radiation. Calculations of economic applicability of the technique have been published many times [55, 56], and the majority of the studies concur in one thing: regardless of a country and insurance scheme, PT is in 2–3 times excess of standard IMRT. However, more thorough analysis of medical expenses including the expenditures for rehabilitation after therapy enabled to conclude that the difference in cost nearly levels, since complication rate after PT is lower, and the assessment of long-term results shows financial advantage [57, 58]. According to estimates, by the year 2023, in European countries there will be 45 centers of proton and ion therapy [59].

In recent years, in Russia the lack in the number of PT centers has started decreasing at a quick rate. By now, over 5 years there has been used modern PT (IMPT) on Russian equipment on the basis of A. Tsyb Medical Radiological Research Centre — Branch of the National Medical Research Radiological Centre (Obninsk, Russia) [60]. There are successfully working centers in Saint Petersburg and Dimitrovgrad. There are plans to establish centers in nearly every federal district. The substantial contribution of Russian researchers to PT technique development is worth mentioning [61–63].

Recently, there have been published several findings of preclinical studies demonstrating proton radiation apart from known physic-dosimetric advantages to have a favorable profile of cellular and biological response of a target and tissues, the expression of genes and proteins; the profile differing markedly from photons [64–67]. In blood plasma of the mice with whole-body proton radiation, transforming growth factor β was significantly higher than that after photon therapy [65]. Moreover, there are data that photon therapy contributes to angiogenesis, in this way enhancing the probability of metastases due to the activation of various pro-angiogenic factors. In contrast, proton radiation, according to the study by Girdhani et al. [66], causes no activation of pro-angiogenic and pro-inflammatory genes, contributes to invasion impairment of *in vitro* tumor cells, decreases tumor growth in mice. By inhibiting integrins and matrix metalloproteinases proton particles significantly reduce invasive and migration properties of tumor cells. Lupu-Plesu et al. [68] in 2017 represented a comparative analysis of the effect proton and photon therapy has on lympho-, angiogenesis, inflammatory, proliferative, as well as immune and anti-tumor responses in squamous cell carcinoma models in HNC. The authors made a univocal conclusion about more favorable effects when using PT. The technique in real clinical practice is likely to have favorable biological properties compared to photon therapy; its advantages are not limited to spatial distribution of a dose and RBE.

## Conclusion

Currently, modern proton therapy is a combination of various technical advances in radiotherapy. It has shown its clinical efficiency in radiation of tumors of varying locations. Proton therapy’s successful usage in HNC therapy is a major contribution to treatment efficiency. The method was instantly included in national recommendations on HNC treatment in different countries [69, 70].

Active scanning technologies, program and technical improvements in dosimetric planning, selection of patients, the application of imaging techniques, new conception of radiobiological properties of protons — all these factors enable proton therapy to have a ranking place in the treatment of tumors of various locations. An increasing number of published prospective studies enable to speak about proton therapy advantages and its important role in current oncology in full agreement with the principles of evidence-based medicine and personalized medicine.

Promising directions are the studies devoted to choosing optimal radiation-sensitizing medical therapy, the combination of proton therapy and immune therapy, as well as a novel technique — FLASH therapy, when is given in an ultrafast mode resulting in multiple reductions of radiation changes in healthy tissues, while a dose in a target remains unchanged.
